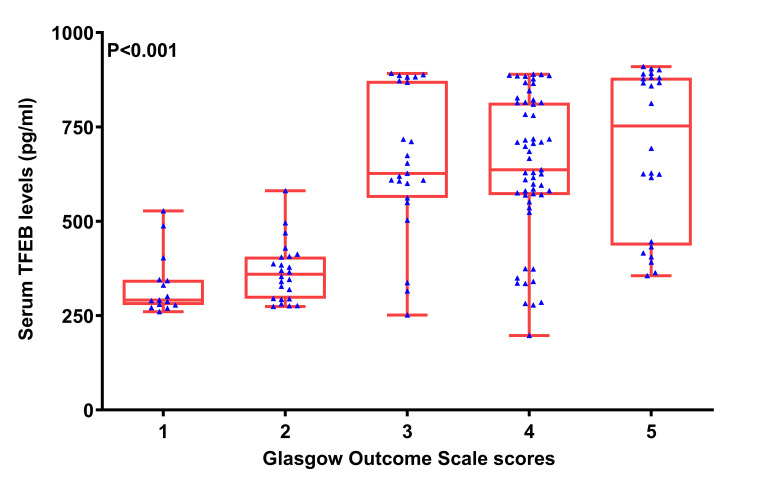# Feasibility of transcription factor EB as a serological metric of poor prognosis following moderate–severe traumatic brain injury: A prospective cohort study

**DOI:** 10.1097/MD.0000000000042271

**Published:** 2025-05-02

**Authors:** Li Zhang, Haiying Ma, Xiaobing Zhang, Yang Zhou, Yuejia Shen, Hefeng Tang, Jiangjuan Shao, Difeng Zhang

**Affiliations:** a Emergency Department, Shaoxing People’s Hospital, Shaoxing, Zhejiang Province, PR China; b Department of Geriatrics, Shaoxing People’s Hospital, Shaoxing, Zhejiang Province, PR China; c Department of Neurosurgery, Shaoxing People’s Hospital, Shaoxing, Zhejiang Province, PR China.

**Keywords:** biomarkers, prognosis, severity, transcription factor EB, traumatic brain injury

## Abstract

Transcription factor EB (TFEB) is an endogenous protective factor. Here, we sought to discern the possibility of serum TFEB as a prognostic biomarker of moderate–severe traumatic brain injury (msTBI). Serum TFEB levels of 141 patients with msTBI and 70 controls were quantified in this prospective cohort study. Rotterdam computed tomography (CT) classification and Glasgow coma scale (GCS) were considered as the severity metrics. Glasgow outcome scale (GOS) scores of 1 to 3 at 6 months after trauma meant a poor prognosis. The results were analyzed using multivariate analysis. Patients versus controls had a notable reduction of serum TFEB levels. Serum TFEB levels of independent correlation with Rotterdam CT scores and GCS scores were independently relevant to continuous GOS scores and ordinal GOS scores. Serum TFEB levels of linear relation to risk of poor prognosis under restricted cubic spline were independently predictive of poor prognosis. Using receiver operating characteristic curve analysis, serum TFEB levels displayed analogous prognostic predictive ability to Rotterdam CT scores and GCS scores. The constructed model by merging the 3 prognostic independent predictors, that is serum TFEB, Rotterdam CT scores and GCS scores, was pictorially exhibited via the nomogram, and was demonstrated to perform well by adopting several statistical approaches. An obvious decline of serum TFEB levels subsequent to msTBI are firmly related to trauma severity and poor neurological outcomes of patients, reinforcing the clinical meaningfulness of serum TFEB as a prognostic biochemical indicator of msTBI.

## 1. Introduction

Moderate–severe traumatic brain injury (msTBI) belongs to a special traumatic form and noticeably features the high mortality and disability rate.^[1]^ External force results in primary brain injury, alongside eliciting secondary brain injury.^[2]^ Resultant molecular mechanisms cover a succession of complex inflammation, oxidation, apoptosis and so forth.^[3]^ Although msTBI prognosis relies on varieties of factors, severity mirrored by Glasgow coma scale (GCS) or Rotterdam computed tomography (CT) classification is virtually acknowledged as the key determinant.^[4]^ Neurological state in activity of daily life, a highly recognized outcome measure of interest in msTBI studies, is frequently assessed via Glasgow outcome scale (GOS).^[5]^ Noteworthy, serological markers, owing to their easy availability, have be rendered with extensive interest as to their clinical contributions to severity estimation and prognosis anticipation during the recent decades.^[6–8]^

Transcription factor EB (TFEB) participates in regulating the expression of autophagy-associated components, thereby enhancing cellular autophagy process.^[9]^ In central nervous system, TFEB may functionally be an endogenous brain-protective agent because it could efficaciously protect brain and spinal cord against acute traumatic, ischemic or hemorrhagic injury via facilitating autophagy.^[10–12]^ Normal cerebral tissues are rich in TFEB.^[13]^ Once suffering from hemorrhagic or ischemic insults, TFEB expressions were notably downregulated in animal brain tissues.^[12,14]^ Also, markedly decreased TFEB levels in brain and serum specimens were firmly correlated with intensity of cognitive impairments in patients with Alzheimer disease.^[15,16]^ As implicated by the above features, serum TFEB may be of clinical usability as a brain injury biomarker. Here, serum TFEB was detected for investigating its prognostic role in msTBI.

## 2. Materials and methods

### 2.1. Study design, participant recruitments, and ethical statement

**Figure 1.**
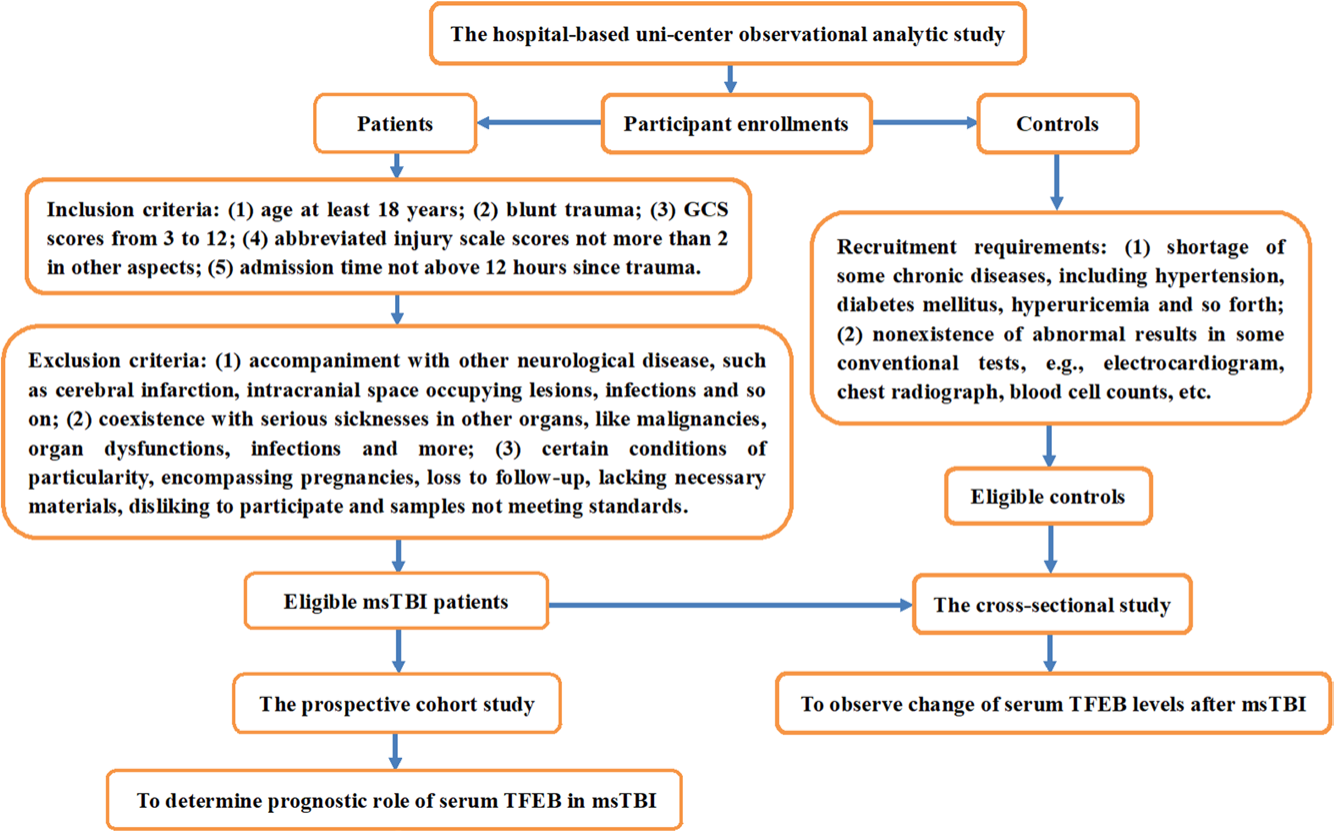
Study plan as regards assessment of biomarker prognostic value in moderate–severe traumatic brain injury. In this observational analytic study encompassing the prospective cohort and cross-sectional sub-studies, controls and patients diagnosed of moderate–severe traumatic brain injury were recruited, so as to observe alteration of serum-based transcription levels and its prognostic value following head trauma. GCS = Glasgow coma scale, msTBI = moderate–severe traumatic brain injury, TFEB = transcription factor EB.

This was a monocenter observational analytic study at the Shaoxing People’s Hospital during the period between May 2020 and July 2023, which was inclusive of 2 sub-studies, that is the cross-sectional analysis and the prospective cohort analysis. In the former, patients with msTBI and controls were enlisted for observing variation of serum TFEB levels after msTBI. In the latter, contribution of serum TFEB to poor prognosis at post-msTBI 6 months was investigated among patients with msTBI. Controls and patients were enrolled in accordance with criteria outlined in Figure 1. The study was implemented by obeying the tenets set forth in the Declaration of Helsinki and the terms formulated by local and institutional laws. The study protocol was given permission from the Ethics Board at the Shaoxing People’s Hospital (approval number: 2021-K-Y-330-01). Legal proxies of patients and controls themselves were acquainted with specific study contents and later independently signed informed consent forms.

### 2.2. Data gatherings, follow-up, and outcome assessment

The documented conventional data consisted of demographics (age and gender), adverse life hobbies (cigarette smoking and alcohol consumption), chronic comorbidities (diabetes, hypertension, and dyslipidemia), and time parameters (post-trauma admission time and sampling time). Traumatic causes were of 2 types: traffic accidence and others. GCS was regarded as a severity metric and the scores were recorded post-resuscitation. At a basis of initial head CT images, some radiologically positive appearances encompassed brain contusion, bleedings in subdural, epidural, subarachnoidal, intraventricular and intracerebral spaces, midline shift distance, and basal cistern statuses. Rotterdam CT scores were computed from the initial radiological images. Via telephone visits, neurological function based on GOS was assessed at posttraumatic 6 months through structured interviews. The scores from 1 to 3 were termed as a poor prognosis.^[17]^

### 2.3. Blood samplings and immune analysis

Five milliliters of venous blood, which was drawn via venipuncture in the antecubital area from all participants, were put in a biochemistry tube for next centrifugation at 2000 × g for 10 minutes. Separated supernatants were moved into Eppendorf tubes so as to get further storage at minus 80 ℃ awaiting final detections. Serum TFEB levels were in batches gauged via a commercially available Enzyme-Linked Immunosorbent Assay kit (catalogue number, MBS9337615; MyBioSource,). The detection sensitivity was 0.1 ng/mL, the detection range spanned from 0.25 ng/mL to 8 ng/mL, and both intra- and inter- assay coefficients of variance were <10%. Following the manufacturer’s manual, serum samples were in duplicate quantified in blinded mode by the same professional person.

### 2.4. Statistical analysis

Here, the SPSS 23.0 (SPSS Inc., Chicago, IL), MedCalc 20 (MedCalc Software, Ltd, Ostend, Belgium), GraphPad Prism 7.01 (GraphPad Software Inc., San Diego, CA), and R 3.5.1 (https://www.r-project.org) were run to make statistical analyses and graphs. Data were classified into 2 large types, namely quantitative and qualitative variables. The former was categorized into continuous and discrete, and the latter was divided into nominal and ordinal. The qualitative variables were summarized as frequencies (percentages), and their two-group and multiple-group comparisons were done by using the chi-square test or Fisher exact test as deemed applicable. As per the Kolmogorov–Smirnov test, normally- and non-normally-distributed quantitative variables were presented as means with standard deviations and medians with lower-upper quartiles separately, and their distinctions between 2 groups and among multiple groups were unveiled by applying the independent-sample Student *t* test, Mann–Whitney *U* test, analysis of variance or Kruskal–Wallis *H* test as considered suitable. Bivariate correlational assessments were finished by employing the Spearman test. Continuous serum TFEB levels, discrete GOS scores, ordinal GOS scores, and binary GOS scores (poor prognosis vs good prognosis) were chosen as 4 dependent variables, and simultaneously the linear regression analysis, binary logistic regression analysis and ordinal regression analysis were selected as 3 multivariate analytic approaches. Factors, which were identified as statistically significant differences on univariate analyses, were given entry into the multivariate models for revelations of independent variables. The restricted cubic spline was plotted for linearity relevance investigation with poor prognosis 6 months following msTBI. The receiver operating characteristic (ROC) curve was graphed for discrimination ability determination. Rotterdam CT scale and GCS was combined to form model 1 and serum TFEB was integrated into model 1 for establishing model 2, in order to unveil whether addition of serum TFEB to model 1 could heighten model 1’s predictive ability for 6-month poor prognosis after msTBI. The nomogram of model 2 was drawn for model visualization. The decision curve and calibration curve were constructed for verifying stability and validity of model 2. The minimum sample size was estimated by GPower 3.1.9.4 (Heinrich-Heine-Universität Düsseldorf, Universitätsstraße 1, Düsseldorf, Germany). The sample size in this study met the requirements. Variances with a two-tailed *P* value < .05 were termed as statistical significance.

## 3. Results

### 3.1. Subject characteristics

An initial enrollment of 192 patients with msTBI was achieved following the pre-established inclusion criteria, a collective of 51 cases were afterwards removed from this study as per the prespecified exclusion criteria, and 141 patients were finally reserved for clinical analysis. Baseline characteristics of all patients were displayed in Table 1. Seventy controls were entered into the clinical study. The controls were aged from 21 to 79 years (mean, 42.6 years; standard deviation, 17.1 years), consisted of 46 males and 24 females, as well as included 15 tobacco smokers and 13 alcohol consumers. Statistically, patients had similar mean age, gender ratio, as well as rates of tobacco smoker and alcohol drinkers (all *P* > .05).

**Table 1:** Baseline characteristics of all patients and bivariate correlation analysis of serum transcription factor EB levels.

	All patients	Spearman test	
		ρ	*P* value
Gender (male/female)	84/57	0.044	.601
Age (years)	42.8 ± 15.5	0.033	.700
Cigarette smoking	47 (33.3%)	-0.075	.374
Alcohol drinking	44 (31.2%)	0.046	.592
Hypertension	26 (18.4%)	-0.050	.557
Diabetes	15 (10.6%)	-0.042	.622
Dyslipidemia	33 (23.4%)	-0.019	.820
Admission time (h)	6.1 (4.0–8.8)	0.108	.202
Sampling time (h)	7.0 (4.7–9.5)	0.110	.196
GCS scores	9 (6–10)	0.549	<.001
Rotterdam CT scores	4 (3–5)	-0.531	<.001
Traffic accidents	68 (48.2%)	-0.114	.177
SAP (mm Hg)	123.6 ± 29.3	0.046	.592
DAP (mm Hg)	77.2 ± 20.2	0.005	.954
Abnormal cisterns	98 (69.5%)	-0.275	.001
Midline shift > 5 mm	80 (56.7%)	-0.228	.007
EDH	66 (46.8%)	-0.093	.271
SDH	84 (59.6%)	-0.150	.075
SAH	95 (67.4%)	-0.120	.158
IVH	10 (7.1%)	-0.092	.280
ICH	66 (46.8%)	-0.046	.587
Brain contusion	81 (57.4%)	-0.068	.423
Blood glucose levels (mmol/L)	9.7 (6.0–11.2)	-0.244	.004
Blood leucocyte count (×10^9^/L)	6.5 (5.1–8.5)	-0.110	.194

Data were reported in form of mean ± standard deviation, median (25–75th percentiles) or count (percentage) as deemed applicable.

CT = computed tomography, DAP = diastolic arterial blood pressure, EDH = epidural hematoma, GCS = Glasgow coma scale, ICH = intracerebral hematoma, IVH = intraventricular hemorrhage, SAH = subarachnoid hemorrhage, SAP = systolic arterial blood pressure, SDH = subdural hematoma.

### 3.2. Serum TFEB levels post-trauma and its pertinence to severity

**Figure 2.**
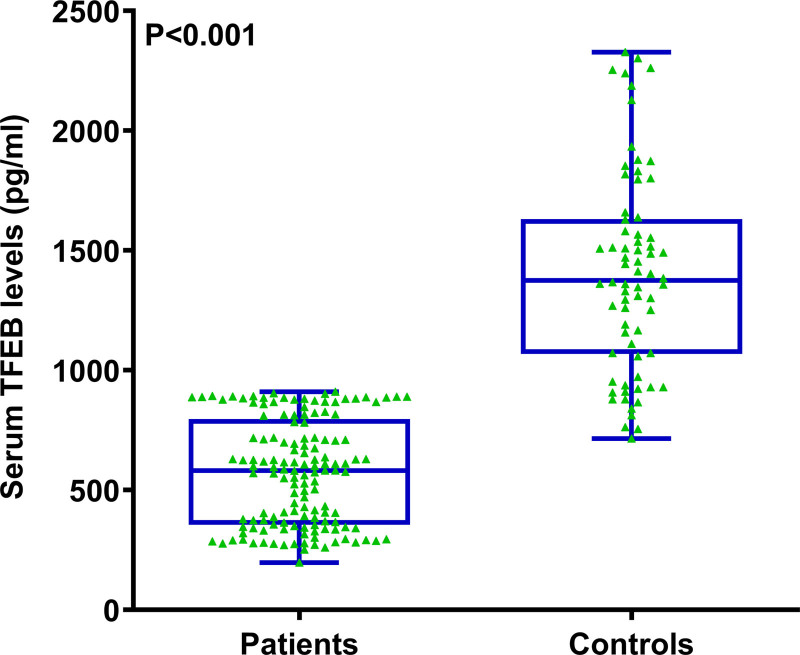
Serum-based transcription factor EB levels following moderate–severe traumatic brain injury. Patients, as opposed to controls, bore extremely decreased serum transcription factor EB levels (*P* < .001). TFEB stands for transcription factor EB.

There was an obvious reduction of serum TFEB levels in patients diseased of msTBI, in contrast to controls (*P* < .001; Fig. 2). Among 10 subgroups with separate GCS scores from 3 to 12, serum TFEB levels were significantly lowest in subgroup with the score 3, the levels were gradually enhanced in other subgroups with the increasing scores from 4 to 11, and the levels were markedly highest in that with the score 12 (*P* < .001; Figure S1, Supplemental Digital Content, https://links.lww.com/MD/O767). Likewise, among 3 subgroups with respective GCS scores 3 to 5, 6 to 8 and 9 to 12, serum TFEB levels were significantly lowest, medium and highest respectively (*P* < .001; Figure S2, Supplemental Digital Content, https://links.lww.com/MD/O768). Similarly, serum TFEB levels were in significantly positive proportion to GCS scores (*P* < .001; Figure S3, Supplemental Digital Content, https://links.lww.com/MD/O769). In addition, serum TFEB levels were significantly highest in patients with Rotterdam CT score 2, followed by the scores 3 to 5, and were substantially lowest in those with the score 6 (*P* < .001; Figure S4, Supplemental Digital Content, https://links.lww.com/MD/O770), as well as displayed evidently opposite connection with the CT scores (*P* < .001; Figure S5, Supplemental Digital Content, https://links.lww.com/MD/O771).

In subsequent correlation analyses with inclusion of other variables, GCS scores and Rotterdam CT scores, along with blood glucose levels, abnormal cisterns and midline shift above 5 mm, were tightly linked to serum TFEB levels (all *P* < .05; Table 1). Taking into account that midline shift and abnormal cisterns composed Rotterdam CT scale, only GCS scores, Rotterdam CT scores, and blood glucose levels were added into the multivariate model. As a result, the factors which were independently correlated with serum TFEB levels consisted of GCS scores [beta (β), 26.995; 95% confidence interval (CI), 13.413–40.577; variance inflation factor (VIF), 1.598; *P* = .001], and Rotterdam CT scores (β, −59.514; 95% CI, −91.417 to 27.611; VIF, 1.551; *P* = .001).

### 3.3. Serum TFEB levels and neurological prognosis indicated by ordinal GOS scores

Among 5 subgroups with separate GOS scores from 1 to 5, subgroup with the score 1 displayed notably lowest serum TFEB levels, followed by other subgroups with the scores from 2 to 4, that with the score 5 exhibited markedly highest serum TFEB levels (*P* < .001; Figure S6, Supplemental Digital Content, https://links.lww.com/MD/O772). Table 2 shows that there were significant disparities in terms of serum TFEB levels, age, GCS scores, Rotterdam CT scores, abnormal cisterns, midline shift more than 5 mm, and blood glucose levels among the 5 groups (all *P* < .05). Given that abnormal cisterns and midline shift belong to the components of Rotterdam CT classification, only the rests were entered into the ordinal logistic regression model. Consequently, serum TFEB levels [odds ratio (OR), 1.003; 95% CI, 1.001–1.005; VIF, 1.632; *P* = .005], GCS scores (OR, 1.347; 95% CI, 1.149–1.579; VIF, 1.810; *P* = .001), and Rotterdam CT scores (OR, 0.641; 95% CI, 0.448–0.919; VIF, 1.948; *P* = .015) remained as the 3 independently associated factors of ordinal GOS scores.

**Table 2:** Baseline features among 5 subgroups at basis of Glasgow outcome scale scores after traumatic brain injury.

	Glasgow outcome scale scores					*P* value
	1	2	3	4	5	
Gender (male/female)	6/9	12/12	15/8	34/21	17/7	.289
Age (years)	55 (48–65)	46 (36–60)	45 (28–50)	35 (26–49)	41 (24–54)	.019
Cigarette smoking	5 (33.5%)	11 (45.8%)	8 (34.8%)	17 (30.9%)	6 (25.0%)	.626
Alcohol drinking	5 (33.5%)	5 (20.8%)	9 (39.1%)	17 (30.9%)	8 (33.3%)	.743
Hypertension	2 (13.3%)	7 (29.2%)	6 (26.1%)	9 (16.4%)	2 (8.3%)	.311
Diabetes	2 (13.3%)	3 (12.5%)	3 (13.0%)	4 (7.3%)	3 (12.5%)	.897
Dyslipidemia	3 (20.0%)	6 (25.0%)	8 (34.8%)	11 (20.0%)	5 (20.8%)	.692
Admission time (h)	3.3 (3.1–9.3)	6.3 (3.7–9.7)	6.2 (4.6–7.7)	6.0 (4.0–8.5)	6.9 (4.5–9.0)	.409
Sampling time (h)	4.0 (3.6–10.2)	7.0 (4.4–10.7)	7.3 (5.2–8.4)	7.0 (5.1–9.4)	8.0 (5.6–9.8)	.432
GCS scores	4 (3–6)	5 (4–7)	9 (8–9)	9 (7–11)	11 (9–12)	<.001
Rotterdam CT scores	5 (4–5)	5 (4–5)	4 (3–5)	3 (3–4)	3 (2–3)	<.001
Traffic accidents	9 (60.0%)	13 (54.2%)	13 (56.5%)	25 (45.5%)	8 (33.3%)	.392
SAP (mm Hg)	103 (82–126)	127 96–160)	125 (120–153)	129 (106–145)	119 (90–133)	.116
DAP (mm Hg)	61 (52–80)	76 (64–99)	77 (70–104)	77 (64–97)	74 (51–94)	.209
Abnormal cisterns	15 (100.0%)	22 (91.7%)	19 (82.6%)	30 (54.5%)	12 (50.0%)	<.001
Midline shift > 5 mm	10 (66.7%)	21 (87.5%)	14 (60.9%)	29 (52.7%)	6 (25.0%)	<.001
EDH	7 (46.7%)	16 (66.7%)	11 (47.8%)	22 (40.0%)	10 (41.7%)	.278
SDH	8 (53.8%)	16 (66.7%)	11 (47.8%)	34 (61.8%)	15 (62.5%)	.688
SAH	10 (66.7%)	19 (79.2%)	15 (65.2%)	36 (65.5%)	15 (62.5%)	.750
IVH	3 (20.0%)	1 (4.2%)	2 (8.7%)	2 (3.6%)	2 (8.3%)	.263
ICH	5 (33.3%)	13 (54.2%)	10 (43.5%)	30 (54.5%)	8 (33.3%)	.309
Brain contusion	9 (60.0%)	15 (62.5%)	11 (47.8%)	34 (61.8%)	12 (50.0%)	.711
Blood glucose levels (mmol/L)	11.5 (9.3–12.5)	10.3 (8.7–12.3)	10.2 (6.3–11.2)	8.9 (5.0–10.5)	8.5 (5.4–10.8)	.031
Blood leucocyte counts (×10^9^/L)	6.2 (5.0–8.3)	6.9 (5.1–10.9)	6.9 (5.4–8.1)	6.0 (5.0–7.4)	7.7 (5.2–9.8)	.164
Serum TFEB levels (pg/mL)	291.1 (278.8–343.8)	359.4 (294.8–405.6)	626.5 (581.0–870.0)	636.6 (569.6–814.1)	752.5 (438.8–880.4)	<.001

All quantitative data were non-normally distributed and subsequently were shown as median (25–75th percentiles). Qualitative data were reported in form of frequency (percentage). The Kruskal–Wallis test and chi-square test were applied for corresponding comparisons.

CT = computed tomography, DAP = diastolic arterial blood pressure, EDH = epidural hematoma, GCS = Glasgow coma scale, ICH = intracerebral hematoma, IVH = intraventricular hemorrhage, SAH = subarachnoid hemorrhage, SAP = systolic arterial blood pressure, SDH = subdural hematoma, TFEB = transcription factor EB.

### 3.4. Serum TFEB levels and neurological prognosis indicated by continuous GOS scores

Serum TFEB levels of patients with msTBI were pronouncedly positively relevant to continuous GOS scores (*P* < .001; Figure S7, Supplemental Digital Content, https://links.lww.com/MD/O773). In next step, besides serum TFEB levels, other variables, that is age, GCS scores, Rotterdam CT scores, blood glucose levels, abnormal cisterns, and midline shift above 5 mm, kept tightly correlated with GOS scores (all *P* < .05; Table 3). Considering abnormal cisterns and midline shift more than 5 mm constituted Rotterdam CT scale, just the remainders were added to the multivariate model. As a consequence, serum TFEB levels (β, 0.002; 95% CI, 0.001–0.003; VIF, 1.614; *P* = .001), GCS scores (β, 0.146; 95% CI, 0.074–0.218; VIF, 1.847; *P* = .001) and Rotterdam CT scores (β, −0.211; 95% CI, −0.376 to 0.045; VIF, 1.723; *P* = .013) had intimate relation to continuous GOS scores.

**Table 3:** Basic features in relation to Glasgow outcome scale scores and poor prognosis after traumatic brain injury.

	Spearman test		Functional outcome		
	ρ	*P* value	Poor prognosis	Good prognosis	*P* value
Gender (male/female)	0.163	.054	33/29	51/28	.174
Age (years)	-0.255	.002	48 (34–60)	37 (26–50)	.003
Cigarette smoking	-0.107	.205	24 (38.7%)	23 (29.1%)	.230
Alcohol drinking	0.031	.718	19 (30.6%)	25 (31.6%)	.899
Hypertension	-0.123	.147	15 (24.2%)	11 (13.9%)	.119
Diabetes	-0.038	.653	8 (12.9%)	7 (8.9%)	.440
Dyslipidemia	-0.046	.585	17 (27.4%)	16 (20.3%)	.318
Admission time (h)	0.103	.225	5.7 (3.3–9.4)	6.2 (4.3–8.8)	.578
Sampling time (h)	0.104	.223	6.6 (4.3–10.0)	7.1 (5.2–9.5)	.546
GCS scores	0.592	<.001	6 (4–9)	9 (8–11)	<.001
Rotterdam CT scores	-0.563	<.001	4 (4–5)	3 (2–4)	<.001
Traffic accidents	-0.163	.054	35 (56.5%)	33 (41.8%)	.083
SAP (mm Hg)	0.021	.802	124 (97–150)	127 (94–143)	.829
DAP (mm Hg)	0.012	.885	73 (61–97)	72 (62–95)	.926
Abnormal cisterns	-0.397	<.001	56 (90.3%)	42 (53.2%)	<.001
Midline shift > 5 mm	-0.337	<.001	45 (72.6%)	35 (44.3%)	.001
EDH	-0.129	.129	34 (54.8%)	32 (40.5%)	.090
SDH	0.038	.653	35 (56.5%)	49 (62.0%)	.503
SAH	-0.075	.375	44 (71.0%)	51 (64.6%)	.420
IVH	-0.083	.328	6 (9.7%)	4 (5.1%)	.289
ICH	-0.015	.856	28 (45.2%)	38 (48.1%)	.728
Brain contusion	-0.034	.693	35 (56.5%)	46 (58.2%)	.832
Blood glucose levels (mmol/L)	-0.239	.004	10.5 (8.5–12.1)	8.9 (5.1–10.5)	.004
Blood leucocyte counts (×10^9^/L)	-0.017	.839	6.9 (5.4–8.8)	6.1 (5.0–8.2)	.257
Serum TFEB levels (pg/mL)	0.545	<.001	385.5 (295.2–606.3)	666.5 (560.3–852.1)	<.001

Data were summarized as mean ± standard deviation, median (25–75th percentiles) or count (percentage) as suitable. Statistical methods covered the Student *t* test, Mann–Whitney *U* test, Pearson chi-square test, and Fisher exact test.

CT = computed tomography, DAP = diastolic arterial blood pressure, EDH = epidural hematoma, GCS = Glasgow coma scale, ICH = intracerebral hematoma, IVH = intraventricular hemorrhage, SAH = subarachnoid hemorrhage, SAP = systolic arterial blood pressure, SDH = subdural hematoma, TFEB = transcription factor EB.

### 3.5. Serum TFEB levels and neurological prognosis indicated by binary GOS scores

**Figure 3.**
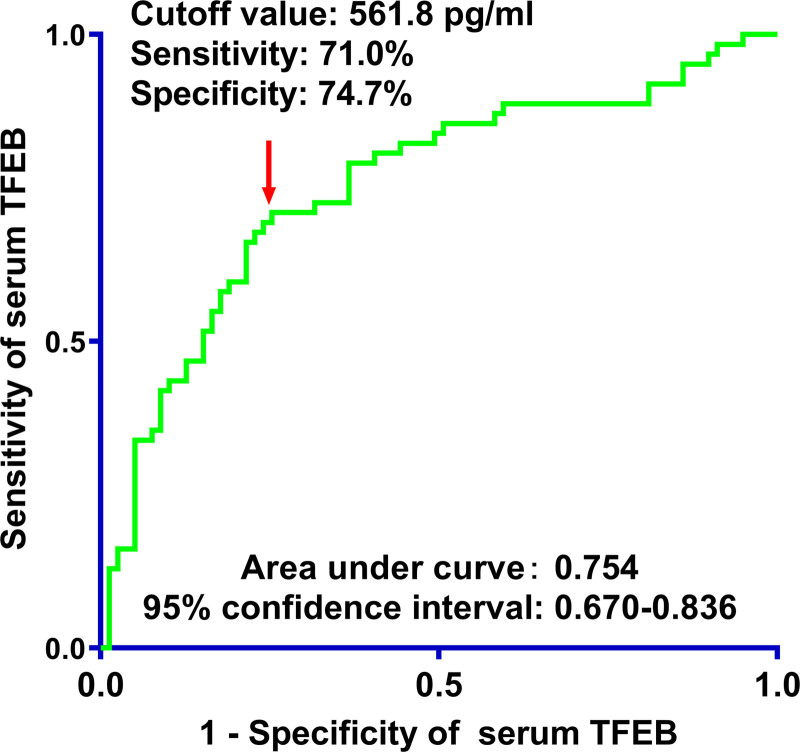
Prognostic effect of serum-based transcription factor EB levels in moderate–severe traumatic brain injury. In the setting of the receiver operating characteristic curve, serum-based transcription factor EB levels were efficiently predictive of poor prognosis following head trauma. Using the Youden method, the threshold value was selected for prognosis anticipation. TFEB = transcription factor EB.

**Figure 4.**
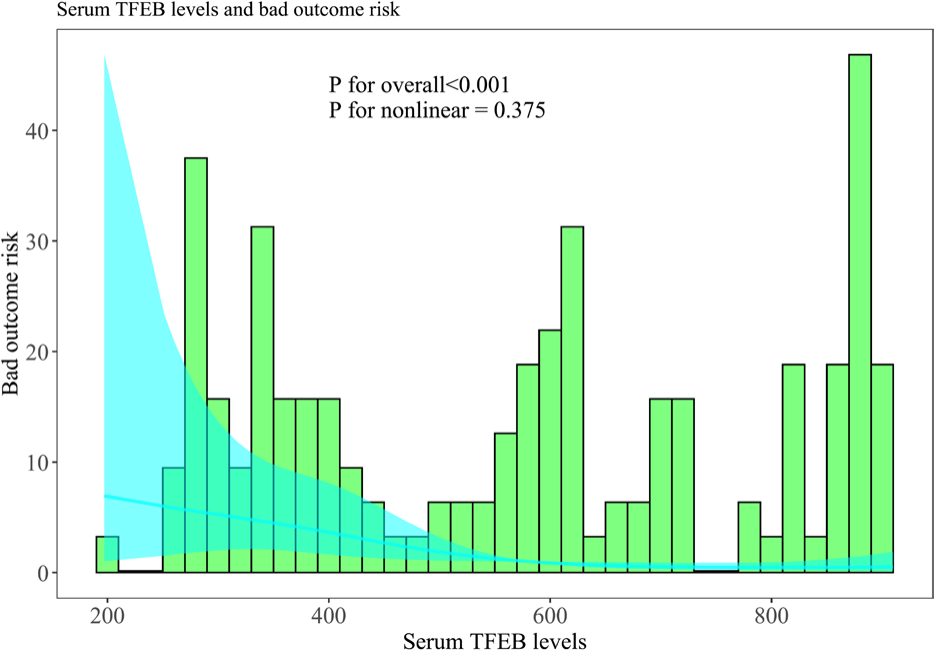
Linearity connection of serum-based transcription factor EB levels with poor prognosis risk after moderate–severe traumatic brain injury. Based on the scenario of the restricted cubic spline, serum-based transcription factor EB levels had linear correlation with poor prognosis likelihood after moderate–severe traumatic brain injury (*P* for nonlinear > .05). TFEB = transcription factor EB.

As described in Figure S8, Supplemental Digital Content, https://links.lww.com/MD/O774, serum TFEB levels were extremely lower in patients with poor prognosis than in those without (*P* < .001). Area under ROC curve at 0.754 was portrayed in Figure 3 for forecasting poor prognosis subsequent to msTBI, wherein the levels below 561.8 pg/mL distinguished probability of poor prognosis with medium-high sensitivity and specificity values. In the following linearity correlational assessment by the restricted cubic spline, serum TFEB levels were revealed to be at linear relation to risk of poor prognosis (*P* for nonlinear > .05; Fig. 4), meaning that serum TFEB levels could be considered as a measurement variable for direct association analysis.

Between patients with poor prognosis and those with good prognosis, serum TFEB levels, coupled with age, GCS scores, Rotterdam CT scores, blood glucose levels, abnormal cisterns, and midline shift > 5 mm, substantially differed (all *P* < .05; Table 3). Because Rotterdam CT classification contained abnormal cisterns in addition to midline shift, the remnant 5 factors were integrated into the multivariate model. Afterwards, it was confirmed that serum TFEB levels (OR, 0.996; 95% CI, 0.994–0.998; VIF, 1.614; *P* = .025), GCS scores (OR, 0.792; 95% CI, 0.654–0.972; VIF, 1.845; *P* = .010), and Rotterdam CT scores (OR, 1.898; 95% CI, 1.172–3.072; VIF, 1.723; *P* = .009) were independently predictive of poor prognosis.

### 3.6. Prognosis model construction based on serum TFEB levels

**Figure 5.**
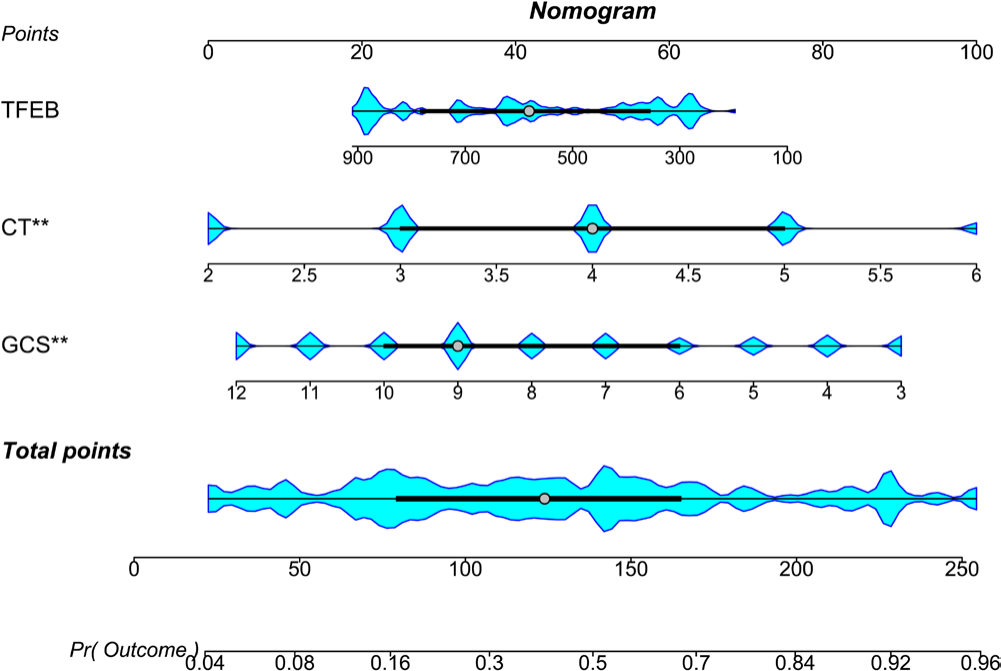
Nomogram visually assessing prognosis model in moderate–severe traumatic brain injury. The independent predictors of poor prognosis, that is, serum transcription factor levels, Glasgow coma scale scores, and Rotterdam computerized tomography scores, were consolidated to build the model for predicting poor prognosis. The model was pictorially represented by the nomogram. CT = computerized tomography, GCS = Glasgow coma scale, TFEB = transcription factor EB.

**Figure 6.**
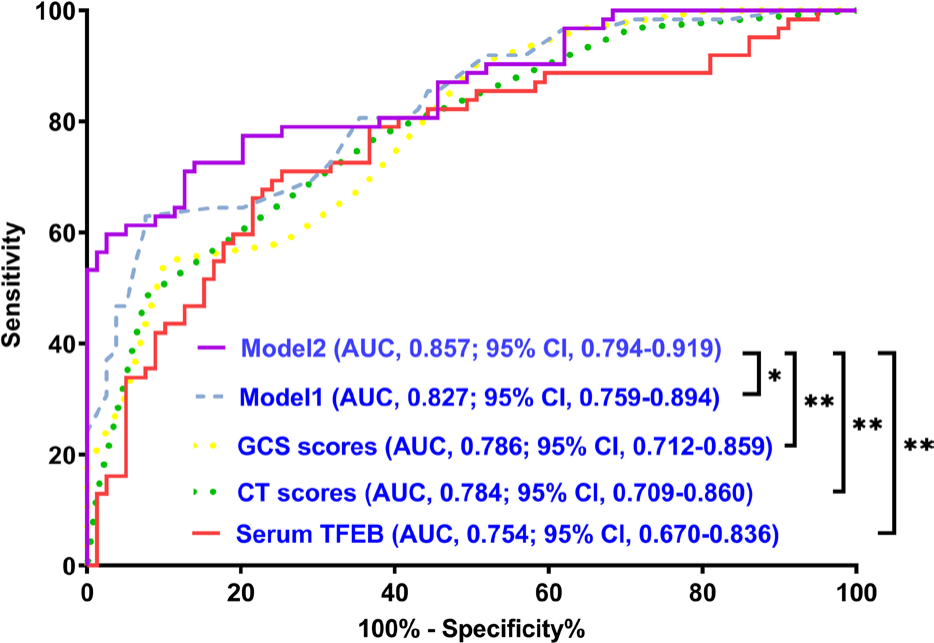
Exhibition of prognosis prediction ability of combined model in moderate–severe traumatic brain injury. Model 1 was composed of serum transcription factor EB levels, Glasgow coma scale scores, and Rotterdam computerized tomography scores. Model 2 comprised Glasgow coma scale scores and Rotterdam computerized tomography scores. In terms of prognosis anticipation, model 1 displayed substantially highest ability among 5 assessment approaches under the receiver operating characteristic curve (^*^P < .05, ^*^P < .01). AUC = area under curve, 95% CI = 95% confidence interval, CT = computed tomography, GCS = Glasgow coma scale, TFEB = transcription factor EB.

**Figure 7.**
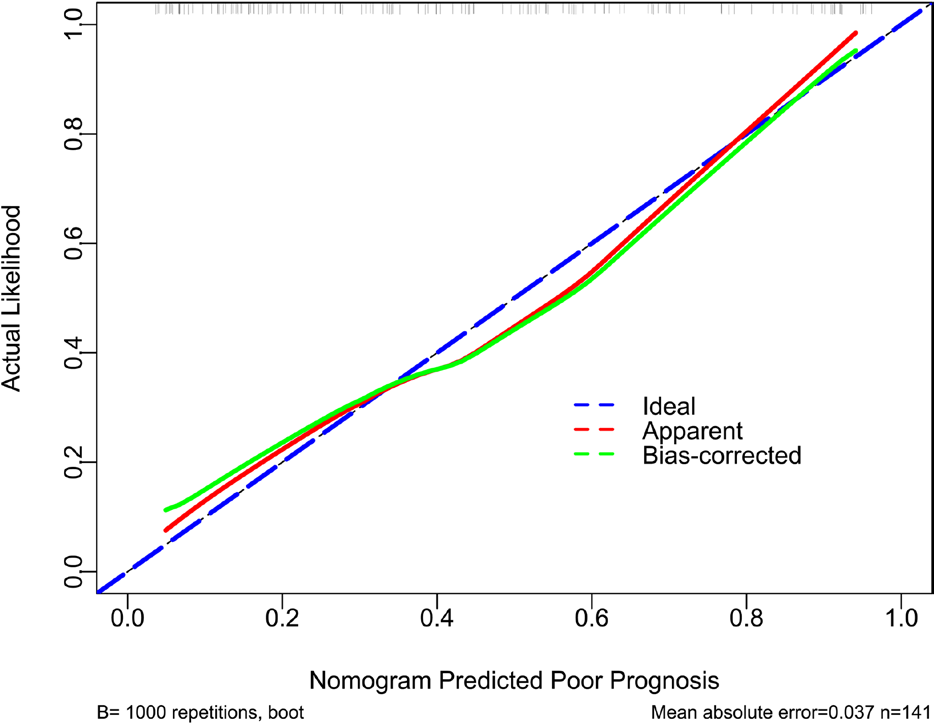
Stability evaluation of prognosis model in moderate–severe traumatic brain injury. Model, in which serum transcription factor EB levels, Glasgow coma scale scores, and Rotterdam computerized tomography scores were integrated, was relatively steady in prognosis prediction in the background of calibration curve.

**Figure 8.**
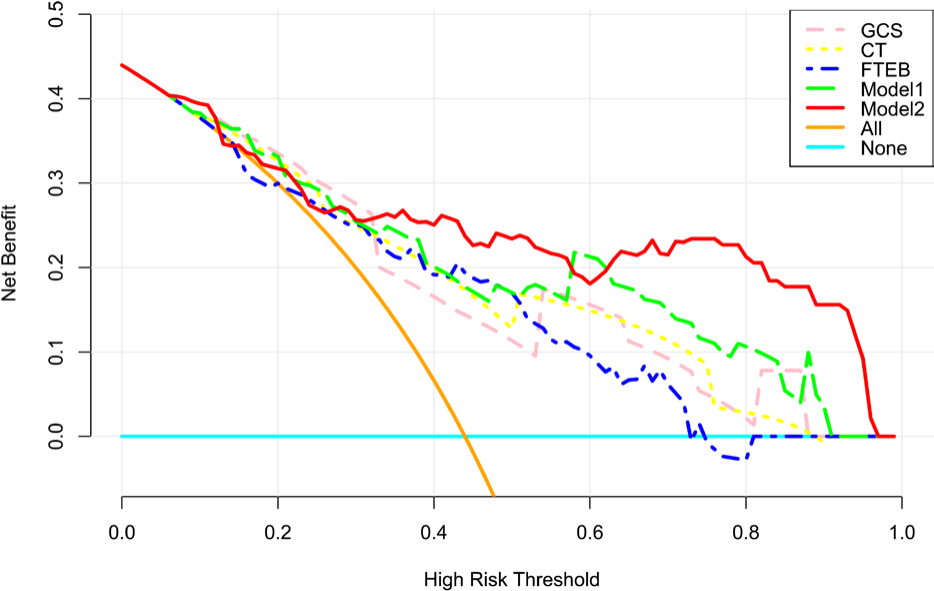
Validity assessment of prognosis model in moderate–severe traumatic brain injury. Model 1 encompassed serum transcription factor EB levels, Glasgow coma scale scores, and Rotterdam computerized tomography scores. Model 2 included Glasgow coma scale scores and Rotterdam computerized tomography scores. In terms of prognosis validity, model 1 displayed significantly highest levels among 5 indicators under the decision curve. CT = computed tomography, GCS = Glasgow coma scale, TFEB = transcription factor EB.

Serum TFEB levels, GCS scores, and Rotterdam CT scores had independent associations with poor prognosis in the current study. GCS scores and Rotterdam CT scores were integrated to create the prognosis model 1. Serum TFEB levels, GCS scores, and Rotterdam CT scores were consolidated to make the prognosis model 2. The model 2 was graphed in form of the nomogram, with the higher total scores corresponding to the higher risk of poor prognosis (Fig. 5). In Figure 6, areas under the ROC curve were found to be numerically, but not statistically different, when serum TFEB levels were in comparison to GCS scores and Rotterdam CT scores (all *P* > .05); and therein, the prognosis model 2 obviously outperformed any of them and even combination of Rotterdam CT scores and GCS scores (model 1) in terms of prognostic predictive power (all *P* < .05). In the stability and validity tests under the calibration curve (Fig. 7) and decision curve (Fig. 8), the model 2 took possession of clinical significance.

## 4. Discussion

As far as we are concerned, this is a first series to perform a measurement of blood TFEB levels in humans after acute brain injury. In the current study, serum TFEB levels were quantified in a group of patients inflicted by msTBI and afterwards some interesting results were found here. Serum TFEB levels were actually promoted after msTBI, were independently correlated with GCS scores and Rotterdam CT scores, as well as were independently associated with neurological function quantified by continuous, ordinal and binary GOS scores at 6-month mark following msTBI. Serum TFEB levels were efficient in predicting poor prognosis after msTBI. Model encompassing 3 independent predictors of poor prognosis, that is GCS scores, Rotterdam CT scores, and serum TFEB levels, had a good performance for predicting poor prognosis. Taken together, serum TFEB may be an encouraging indicator of severity evaluation and prognosis anticipation in patients with msTBI.

It has been fully confirmed that TFEB may be an endogenous protective factor in central nervous system diseases. Specifically, TFEB overexpression could obviously promote cellular autophagy of cerebral cortices, repress neuronal apoptosis, attenuate brain edema, and therefore recover neurological function in rats with experimental subarachnoid hemorrhage.^[12]^ Likewise, by applying neuron-specific overexpression and knockdown techniques, TFEB was demonstrated to bear protective properties against rat brain damage from permanent middle cerebral artery occlusion via restoring dysregulation of the autophagy–lysosomal pathway.^[18]^ Also, in a murine model with traumatic spinal cord injury, inhibition of TFEB led to inverse effects, that is enhancement in pyroptosis and oxidative stress.^[10]^ Collectively, TFEB could become a therapeutic agent of acute brain injury disorders.

TFEB expressions in brains subjected to subarachnoid hemorrhage was significantly diminished in rats endovascularly perforated.^[12]^ Following cerebral ischemia–reperfusion injury in rats, brain TFEB expressions was also decreased markedly.^[14]^ Intriguingly, human brains with Alzheimer disease and amyotrophic lateral sclerosis, relative to normal brains, had notably reduced TFEB expression levels.^[15]^ Alternatively, as opposed to cognitively-unimpaired individuals, patients diagnosed of Alzheimer disease had an exhibition of significantly declined serum TFEB levels.^[16]^ Consistently, serum-based TFEB levels were substantially lower in patients diseased of msTBI than in controls here. The accumulating data are indicative of the notion that blood TFEB levels may be reduced following msTBI. On account of TFEB acting as an endogenous protective factor,^[10–12]^ it is postulated that blood TFEB may be consumed in response to acute brain injury, therefore leading to a reduction of serum TFEB levels after msTBI. From other perspective, TFEB supplementation may be an alternative choice for treating secondary brain injury following msTBI.

Severity assessment is of paramount importance in management of TBI. Conventionally, clinicians favor use of GCS and Rotterdam CT classification in clinical work of TBI.^[19,20]^ The 2 indices were applied as severity assessment indicators in the present study. As for univariate analysis, GCS was considered as the 3 statues, that is continuous variable, ordinal variable and ternary variable; and Rotterdam CT scale was deemed as the 2 states, namely continuous and ordinal variables. The final results were that serum TFEB levels were significantly lower in patients with lower GCS scores or in those with higher Rotterdam CT scores. Furthermore, serum TFEB levels were correlated with Rotterdam CT scores and GCS scores independent of other confounding variables. These data solidify serum TFEB as a potential severity assessment metric of human msTBI.

GCS and Rotterdam CT classification are the 2 common determinants of poor prognosis among TBI patients.^[19,20]^ GOS is preferred for assessing functional outcome after TBI.^[21,22]^ In the study, GOS was identified as the ordinal, continuous and binary variables for reflecting neurological function of msTBI. Here, 3 multivariate models, that is, the binary logistic regression model, ordinal regression model and linear regression model, were operated so that it was verified that serum levels, along with GCS scores and Rotterdam CT scores, appeared as the 3 factors, which were independently associated with poor neurological function of patients suffering from msTBI. Here, serum TFEB levels efficaciously differentiated between patients with poor prognosis and those with good prognosis with area under the ROC curve at 0.754. For the sake of prognostic value of serum TFEB levels, its comparison with GCS scores and Rotterdam CT scores were done, wherein negligible distinctions were revealed. Moreover, 2 models were constructed. One encompassed GCS scores, Rotterdam CT scores, and serum TFEB levels. The other comprised GCS scores and Rotterdam CT scores. Combined model of 3 components displayed statistically significantly highest prognostic ability among all designated 5 parameters, that is GCS scores, serum TFEB levels, Rotterdam CT scores, and the 2 models. Alternatively, the combined model was pictorially delineated by a nomogram. The comparative validity and stability were proved of the combined model in the help of decision curve and calibration curve. So, serum TFEB showed clinical significance in prognosis anticipation by means of model construction. Overall, serum TFEB may be a potential prognosticator of poor prognosis in the scenario of msTBI.

In order to analyze relationship between serum TFEB levels, severity and neurological prognosis after msTBI, this study contained 2 sub-studies, that is the cross-sectional and prospective cohort studies. The former was designed to investigate relation of serum TFEB levels with severity reflected by GCS scores and Rotterdam CT scores. The latter was implemented to determine association of serum TFEB levels with neurological prognosis mirrored by GOS scores at 6 months following msTBI. All severity and prognosis associations were assessed using univariate analyses followed by multivariate analyses, wherein significantly different variables on univariate analyses were incorporated into the multivariate models for identifying independent factors, therefore ensuring enough robustness of study results.

Here, 3 data forms were applied of GOS so as to evaluate neurological prognosis of patients, which encompassed continuous, ordinal and binary types of GOS. From the statistical view, the causal relationship may not be determined by applying the Spearman test followed by the multivariate linear regression analysis, and the multivariate ordinal regression analysis may be characterized by a small sample size in multiple subgroups. For the sake of compensating for the 2 shortcomings mentioned above, the GOS was dichotomized into the 2 conditions (poor prognosis vs good prognosis). Subsequently, the binary logistic regression analysis was employed, so that serum TFEB levels, GCS scores, and Rotterdam CT scores were verified to be the 3 independent predictors of poor prognosis. Hence, close association of serum TFEB levels with poor prognosis of msTBI was sufficiently confirmed from other perspective. Admittedly, increasing sample size can further heighten reliability and stability of results.

Taking into account that GCS and Rotterdam CT classification are the 2 conventional severity metrics of TBI,^[4,19,20]^ the 2 indicators were combined to establish a model, as named as model 1 in this study, for prognosis anticipation. In addition, serum TFEB was added to the preceding model 1 to validate whether addition of serum TFEB could enhance prognostic predictive ability of the above-mentioned model 1. The statistical finding is that serum TFEB combined with GCS and Rotterdam CT classification, as called as model 2 in the current study, may actually promote predictive ability for 6-month poor prognosis of msTBI patients. Thus, serum TFEB, from other aspect, was demonstrated to possibly take possession of effective prognostic prediction ability in the scenario of msTBI.

In the present study, blood glucose levels were substantially associated with poor prognosis in the framework of univariate analysis, but this association was not existent by applying multivariate analysis. This sort of unstable association in our study may be explained by such a phenomenon that blood samples were collected at admission of these patients and therefore blood glucose levels may be variable at non-fasting state, affected by several factors, including stress response, brain injury stimulation, and dietary impact,^[23]^ thereby leading to an inconsistence of study results. Moreover, too high or too low blood glucose levels following TBI negatively affect patient prognosis,^[23]^ indicating close relation of blood glucose levels in form of continuous variable to poor prognosis of TBI may be easily weakened in this condition. In this sense, although blood glucose levels are frequently reported to be independently associated with clinical outcomes of TBI,^[24,25]^ several substitutive indicators, such as serum glucose and potassium ratio, serum glucose–phosphate ratio, and triglyceride glucose index, have been developed to be connected with clinical outcomes of TBI.^[26–30]^ Certainly, inconsistencies in conclusions regarding intimate relationship between blood glucose levels and poor prognosis of TBI may be eliminated by increasing sample size in future.

There are several strengths and weaknesses here. The strengths are that (1) this study, as far as we are aware, is the first investigation for determining prognostic role of serum TFEB in a group of msTBI patients and thereafter found that serum TFEB may be a useful biomarker of severity assessment and prognosis prediction in human msTBI; (2) in order to amply substantiate the conclusion that serum TFEB may be of clinical value as prognostic biomarker, both severity correlation and prognosis relevance were demonstrated using numerous multivariable models. The weaknesses are that (1) from statistical view, 141 patients are of enough power in clinical analyses. However, the study was conducted in a single center and lacked multicenter validation, and there may be regional and sample selection biases. Thus, the validation of conclusions in larger-scale cohorts are obligatory preceding clinical application; (2) temporal trajectory of serum TFEB levels post-msTBI were not explored here, and assessment of serum TFEB levels at multiple time points postadmission would be an imperative task because this would facilitate the observation of the dynamic changes in serum TFEB levels throughout msTBI so as to proffer some valuable information; (3) given that this is an observational clinical study, the direct association between TFEB levels and clinical efficacy has not been explored in depth; so the potential of TFEB as a therapeutic target can be further explored in future via a series of basic experiments and subsequent clinical translational researches.

## 5. Conclusions

In this cohort of patients inflicted by msTBI, a marked decline of serum TFEB levels is independently related to trauma severity and poor 6-month prognosis; and serum TFEB levels have an exhibition of efficacious prognostic capability, and serum TFEB combined with GCS and Rotterdam CT scale displays more satisfactory predictive ability for poor 6-month prognosis, reinforcing TFEB as a serological prognostic metric in msTBI.

## Acknowledgements

We gratefully thank all study participants, their relatives, and the staffs at the recruitment centers for their invaluable contributions.

## Author contributions

**Conceptualization:** Li Zhang.

**Data curation:** Haiying Ma, Yuejia Shen, Hefeng Tang, Jiangjuan Shao, Difeng Zhang.

**Formal analysis:** Haiying Ma, Xiaobing Zhang, Yuejia Shen, Hefeng Tang, Jiangjuan Shao.

**Investigation:** Xiaobing Zhang, Yang Zhou, Yuejia Shen, Hefeng Tang, Difeng Zhang.

**Methodology:** Xiaobing Zhang, Yang Zhou, Difeng Zhang.

**Supervision:** Yang Zhou, Jiangjuan Shao, Difeng Zhang.

**Software:** Yuejia Shen, Hefeng Tang, Difeng Zhang.

**Visualization:** Yuejia Shen, Hefeng Tang.

**Writing – original draft:** Li Zhang, Haiying Ma, Yang Zhou, Hefeng Tang, Jiangjuan Shao, Difeng Zhang.

**Writing – review & editing:** Li Zhang, Haiying Ma, Xiaobing Zhang.

## Supplementary Material



**Figure:**