# Supplementary material for: Feasibility of transcription factor EB as a serological metric of poor prognosis following moderate–severe traumatic brain injury: A prospective cohort study
Source: Medicine (Baltimore). 2025 May 2;104(18):e42271. doi: 10.1097/MD.0000000000042271 (PMC12055063; doi:10.1097/MD.0000000000042271)

**Supplemental Figure 1**

Serum transcription factor EB levels among subgroups with distinct Glasgow coma scale scores following moderate-severe traumatic brain injury.

Serum transcription factor EB levels were substantially raised among subgroups marked in order of Glasgow coma scale scores from 3 to 12 (P<0.001).

TFEB stand for transcription factor EB.


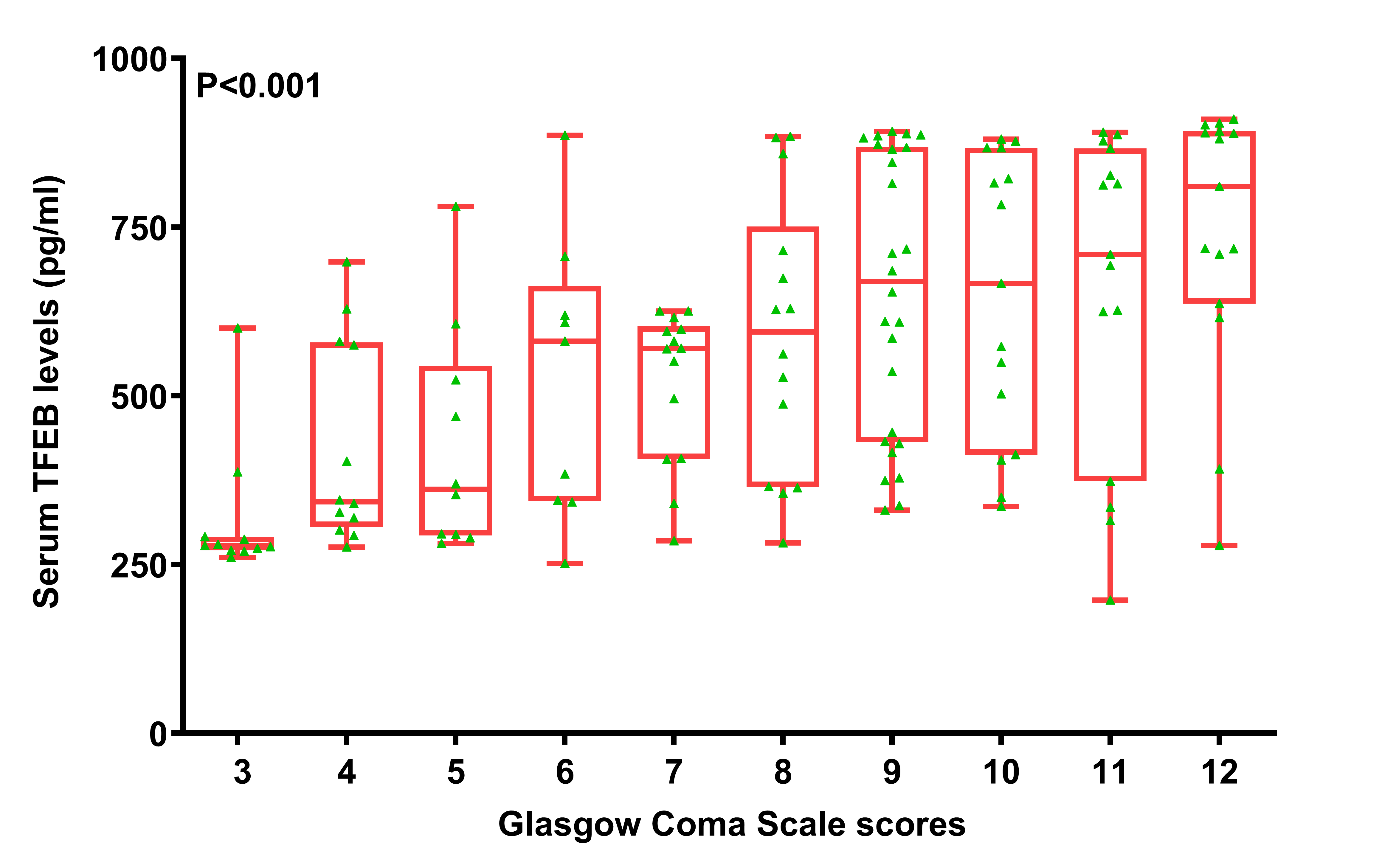

Supplement: Supplementary file 1 [file medi-104-e42271-s001.docx]
