# Supplementary material for: Feasibility of transcription factor EB as a serological metric of poor prognosis following moderate–severe traumatic brain injury: A prospective cohort study
Source: Medicine (Baltimore). 2025 May 2;104(18):e42271. doi: 10.1097/MD.0000000000042271 (PMC12055063; doi:10.1097/MD.0000000000042271)

**Supplemental Figure 2**

Serum transcription factor EB levels among patients grouped as per Glasgow coma scale scores subsequent to moderate-severe traumatic brain injury.

Patients with Glasgow coma scale scores 3-5 held significantly lowest serum transcription factor EB levels; those with the scores 6-8, the medium levels; and those with the scores 9-12, the substantially highest levels (P<0.001).

GCS indicates Glasgow coma scale; TFEB, transcription factor EB.


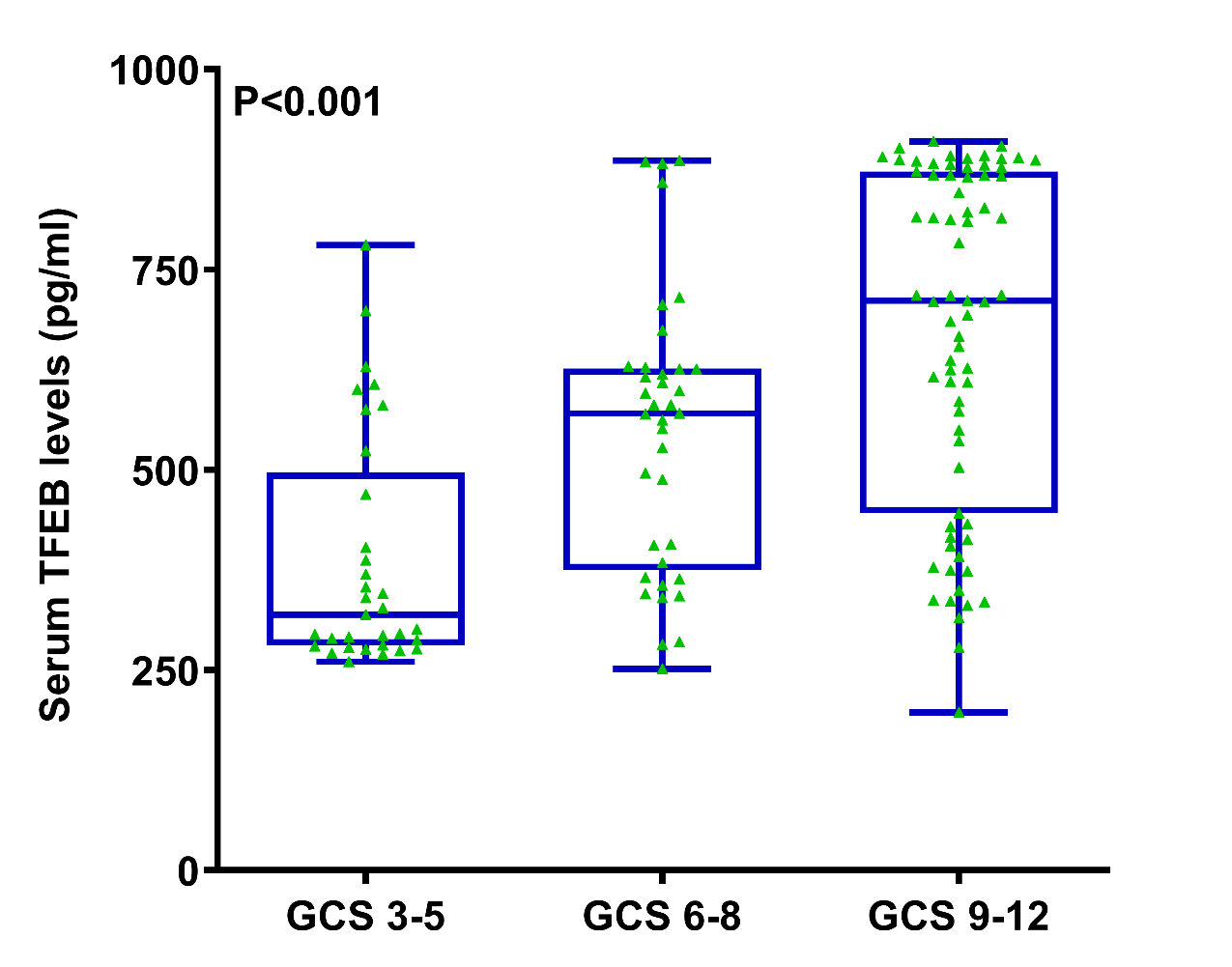

Supplement: Supplementary file 2 [file medi-104-e42271-s002.docx]
