# Supplementary material for: Feasibility of transcription factor EB as a serological metric of poor prognosis following moderate–severe traumatic brain injury: A prospective cohort study
Source: Medicine (Baltimore). 2025 May 2;104(18):e42271. doi: 10.1097/MD.0000000000042271 (PMC12055063; doi:10.1097/MD.0000000000042271)

**Supplemental Figure 3**

Serum transcription factor EB levels and Glasgow coma scale scores after moderate-severe traumatic brain injury.

Serum transcription factor EB levels were strongly correlated with Glasgow Coma Scale scores among patients suffering from moderate-to-severe traumatic brain injury (P<0.001).

GCS denotes Glasgow coma scale; TFEB, transcription factor EB.


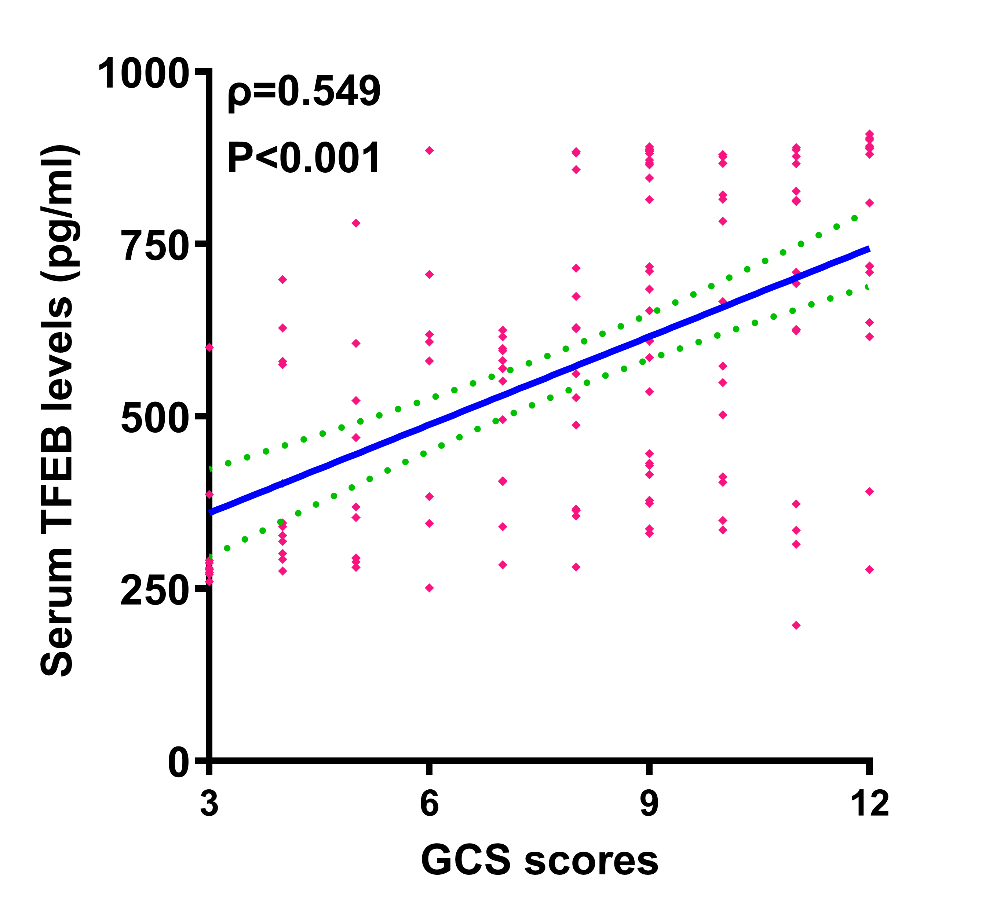

Supplement: Supplementary file 3 [file medi-104-e42271-s003.docx]
