# Supplementary material for: Feasibility of transcription factor EB as a serological metric of poor prognosis following moderate–severe traumatic brain injury: A prospective cohort study
Source: Medicine (Baltimore). 2025 May 2;104(18):e42271. doi: 10.1097/MD.0000000000042271 (PMC12055063; doi:10.1097/MD.0000000000042271)

**Supplemental Figure 4**

Serum transcription factor EB levels among patients divided in accordance with Rotterdam computed tomography scores following moderate-severe traumatic brain injury.

Serum transcription factor EB levels were notably reduced in order of Rotterdam computed tomography scores from 2 to 6 among patients with moderate-severe traumatic brain injury (P<0.001).

CT means computed tomography; TFEB, transcription factor EB.


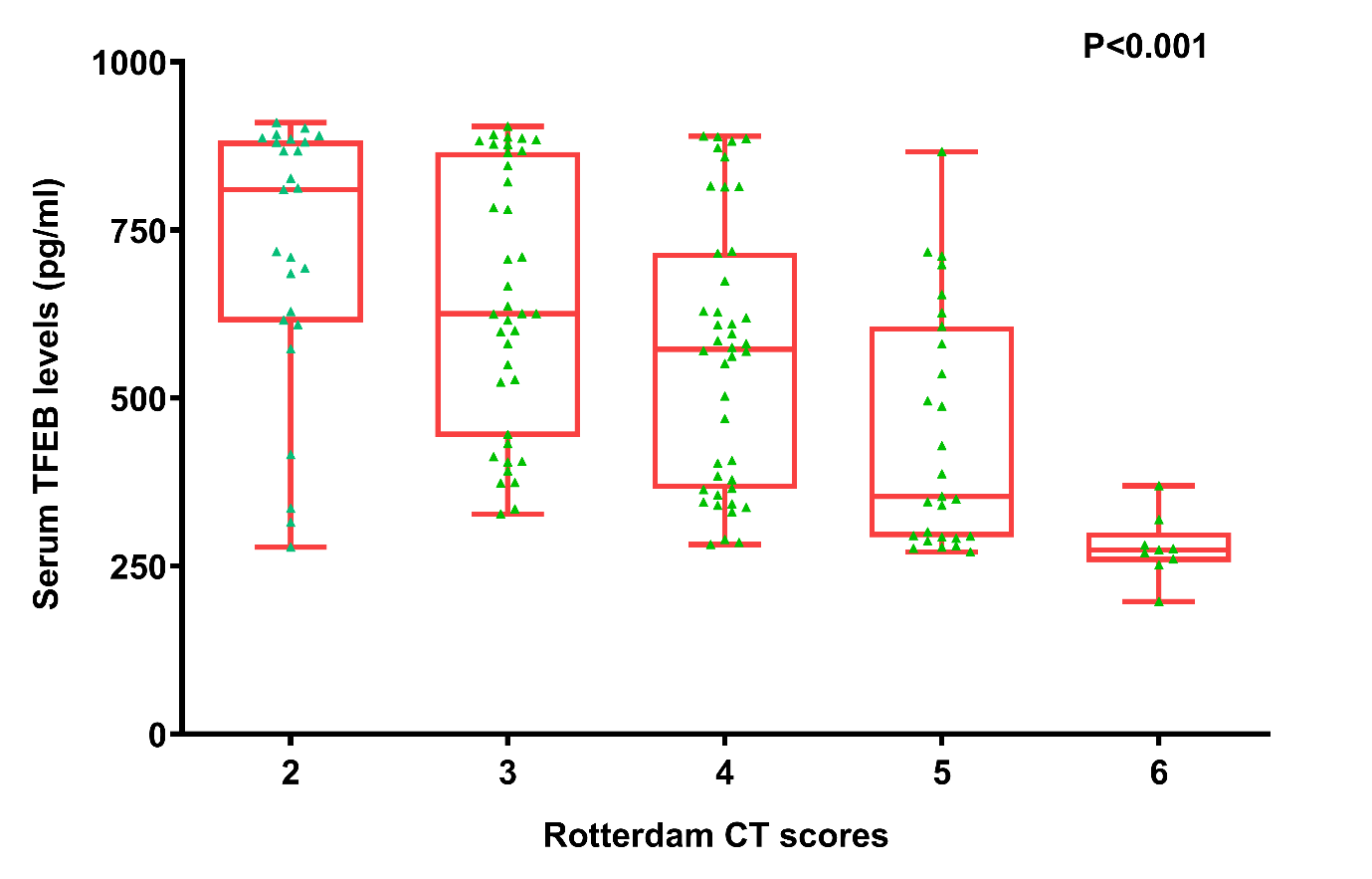

Supplement: Supplementary file 4 [file medi-104-e42271-s004.docx]
