# Supplementary material for: Feasibility of transcription factor EB as a serological metric of poor prognosis following moderate–severe traumatic brain injury: A prospective cohort study
Source: Medicine (Baltimore). 2025 May 2;104(18):e42271. doi: 10.1097/MD.0000000000042271 (PMC12055063; doi:10.1097/MD.0000000000042271)

**Supplemental Figure 5**

Serum transcription factor EB levels and Rotterdam computed tomography scores following moderate-severe traumatic brain injury.

Serum transcription factor EB levels were in inverse proportion to Rotterdam computed tomography scores after moderate-to-severe traumatic brain injury (P<0.001).

CT signifies computed tomography; TFEB, transcription factor EB.


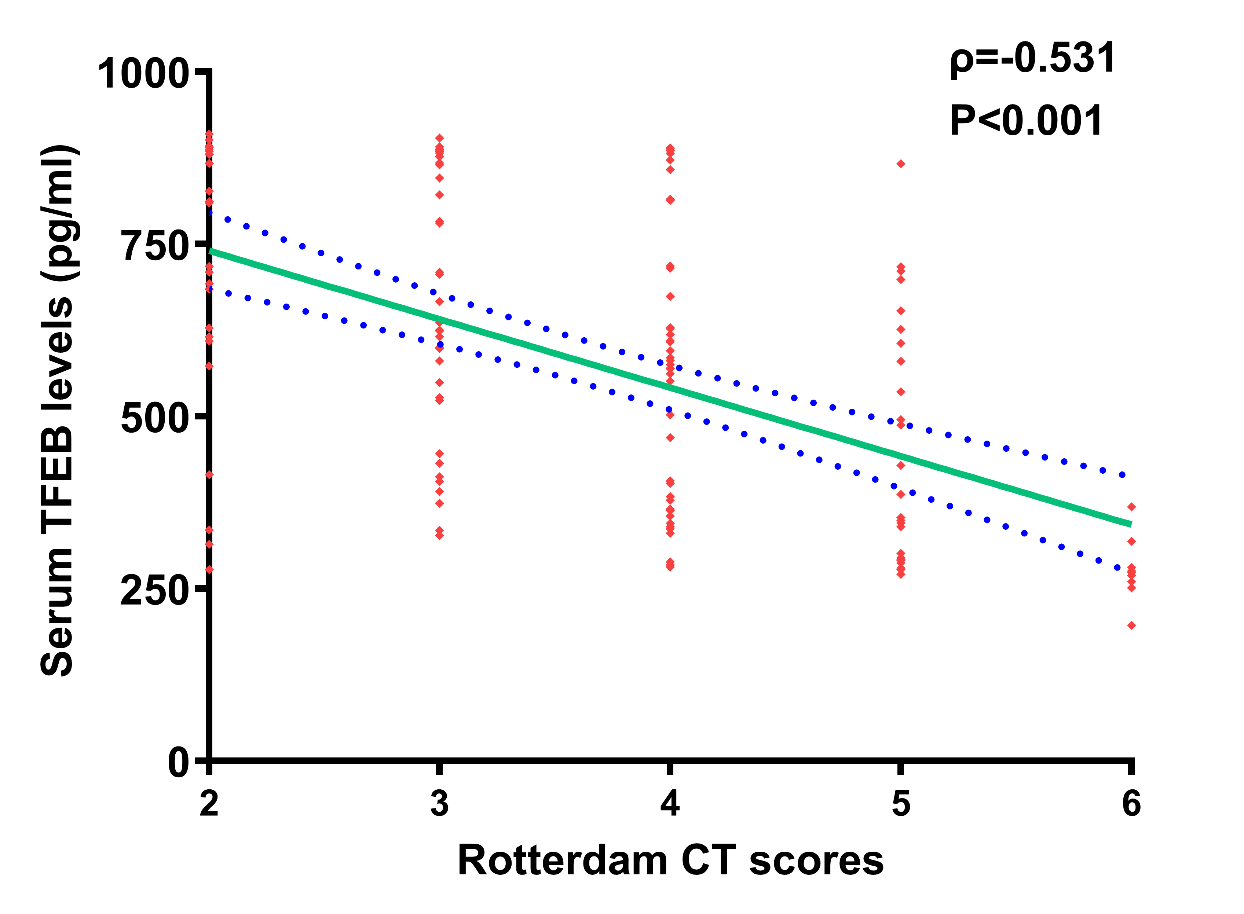

Supplement: Supplementary file 5 [file medi-104-e42271-s005.docx]
