# Supplementary material for: Feasibility of transcription factor EB as a serological metric of poor prognosis following moderate–severe traumatic brain injury: A prospective cohort study
Source: Medicine (Baltimore). 2025 May 2;104(18):e42271. doi: 10.1097/MD.0000000000042271 (PMC12055063; doi:10.1097/MD.0000000000042271)

**Supplemental Figure 8**

Serum transcription factor EB levels between patients with poor prognosis and those with good prognosis subsequent to moderate-severe traumatic brain injury.

Glasgow outcome scale scores 1-3 and 4-5 were termed as poor prognosis and good prognosis separately. Transcription factor EB levels were substantially lower in patients with poor prognosis than in those with good prognosis (P<0.001).

GOS signifies Glasgow outcome scale; TFEB, transcription factor EB.


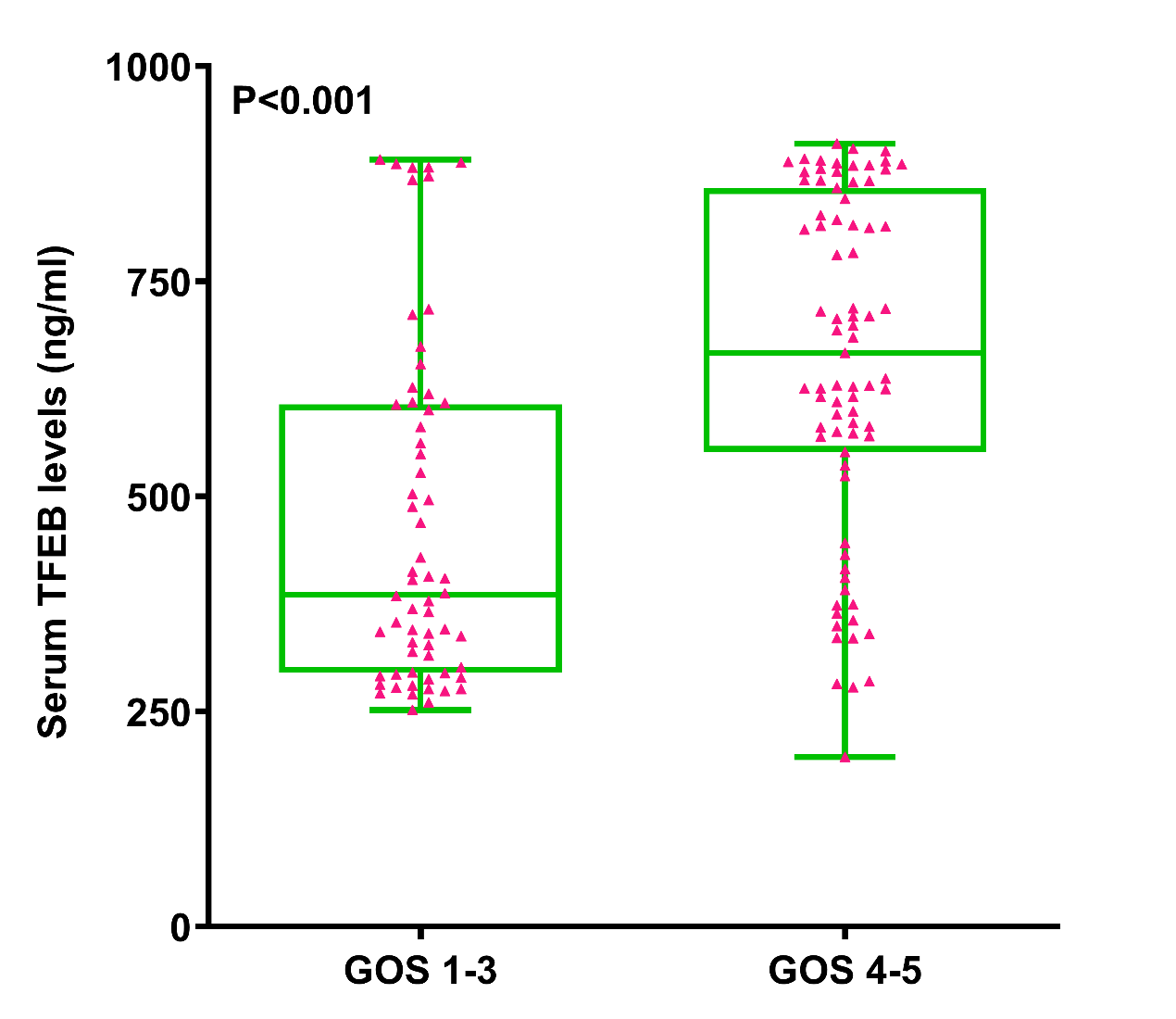

Supplement: Supplementary file 7 [file medi-104-e42271-s008.docx]
